# Characteristics and prognostic factors of COVID-19 among infected cases: a nationwide Tunisian analysis

**DOI:** 10.1186/s12879-021-05844-y

**Published:** 2021-02-03

**Authors:** Chahida Harizi, Ines Cherif, Nourhene Najar, Molka Osman, Rym Mallekh, Oumaima Ben Ayed, Yosr Ayedi, Sonia Dhaouadi, Aicha Hchaichi, Mouna Safer, Hejer Letaief, Ilhem Bouaziz, Sondes Derouiche, Donia Gharbi, Leila Bouabid, Souha Bougatef, Hamida Ben Salah, Radhouane Fakhfakh, Salma Abid, Ilhem Boutiba Ben Boubaker, Mohamed Kouni Chahed, Nissaf Bouafif Ben-Alaya

**Affiliations:** 1 Department of Epidemiology and Statistics, Abderrahman Mami Hospital, Ariana, Tunisia; 2 Faculty of Medicine of Tunis, University of Tunis El Manar, Tunis, Tunisia; 3 National Observatory of New and Emerging Diseases of Tunisia, Tunis, Tunisia; 4 Charles Nicolle Hospital, Laboratory of Microbiology, Virology Unit, National Influenza and other Respiratory Viruses Center-Tunisia, Tunis, Tunisia; 5 Department of Epidemiology and Public Health, Faculty of Medicine of Tunis, Tunis El-Manar University, Tunis, Tunisia

**Keywords:** COVID-19, SARS CoV-2, Prognostic factors, Clinical characteristics

## Abstract

**Background:**

The outbreak of coronavirus disease (COVID-19) continues to constitute an international public health concern. Few data are available on the duration and prognostic factors of the disease. We aimed to study the recovery time among a Tunisian cohort of COVID-19 confirmed patients and identify the prognostic factors.

**Methods:**

A retrospective, nationwide study was conducted from March 2 to May 8, 2020, recruiting all patients who were diagnosed with COVID-19, by RT-PCR methods, in Tunisia. Data were collected via phone call interview. Kaplan-Meir Methods and Cox proportional hazards regression models were, respectively, used to study the recovery time and estimate its prognostic factors.

**Results:**

One thousand and thirty patients with COVID-19 (aged 43.2 ± 18.2 years, 526 female (51.1%)) were enrolled. Among them 141 (14.8%) were healthcare professionals. Out of 173 patients (17.8%) admitted to the hospital, 47 were admitted in an intensive care unit. Among 827 patients who didn’t require specialized care, 55.5% were self-isolated at home, while the rest were in specialized centers. Six hundred and two patients were symptomatic. A total of 634 (61.6%) patients have recovered and 45 (4.4%) patients died. The median duration of illness was estimated to be 31 days (95% CI: [29–32]). Older age (HR = 0.66, CI:[0.46–0.96], *P* = 0.031) and symptoms (HR = 0.61, CI:[0.43–0.81], *P* = 0.021) were independently associated with a delay in recovery time. Being a healthcare professional (HR = 1.52, CI: [1.10–2.08], *P* = 0.011) and patients in home isolation compared to isolation centers (HR = 2.99, CI: [1.85–4.83], *P* < 10¯^3^) were independently associated with faster recovery time.

**Conclusion:**

The duration of illness was estimated to be 1 month. However, this long estimated duration of illness may not equate to infectiousness. A particular attention must to be paid to elderly and symptomatic patients with closer monitoring.

## Background

Coronavirus disease 2019 (COVID-19), an infection caused by severe acute respiratory syndrome coronavirus-2 (SARS-CoV-2), was first reported in December 2019, in Wuhan, Hubei Province in China [1]. Since then, human -to-human transmission rates across the world has soared dramatically making it one of the most challenging global health problems. On 30 January 2020, the World health organization considered COVID-19 as a public health emergency of international concern and announced the pandemic threat in March 11,2020 [1, 2] . On May 15, 2020, 4,553,394 confirmed cases of covid-19 were identified, worldwide, with death rate of 6.7 and 37.7% of recovery [3].

In Tunisia, the first confirmed case of COVID-19 was reported on March 2, 2020. Since then, several measures have been taken to break the spread of the epidemic among the population. These steps include the successive screening at point of entry, 14 days isolation of travelers returning from risk areas, closure of school and university facilities, ban of sports and cultural events, borders closure, curfew and finally, a national lockdown was announced on March 22, 2020 [4]. On May 8, 2020, the total number of confirmed cases of COVID-19 was 1030 [5].

Several studies have addressed clinical, radiological and biological issues in COVID-19 patients [6–10]. The clinical spectrum is large and ranges from asymptomatic or mild forms, accounting for about 80%, to cases of acute respiratory distress syndrome that may lead to intensive care or death [11].

However, we note a scarcity of data on prognostic factors associated with a better outcome and recovery. Since an early recovery reduces the shedding and spread of the virus, identifying recovery prognostic factors may improve our understanding of the dynamics of transmission and foster the control of the epidemic [12].

The present study aims to estimate recovery time and identify related prognostic factors among Tunisian infected people with COVID-19.

## Methods

### Study design and population

This was a retrospective nationwide cohort study. It included all confirmed COVID 19 cases listed among the database of the national observatory of new and emerging diseases (ONMNE), which is the national center for disease control and surveillance, from March 2 to May 8, 2020.

### Case definition

A confirmed COVID-19 case was defined as any person, whether symptomatic or not, with laboratory confirmation of SARS-CoV-2 infection using real-time reverse transcriptase–polymerase chain reaction (RT-PCR).

### Recovery monitoring and criteria

From March 2, 2020, when the first case was reported, all confirmed cases has been consistently and timely reported by the laboratory to the national observatory for new and emerging diseases (ONMNE).

All positive cases were isolated for at least 14 days, at home or in dedicated COVID-19 centers, and were not released until they got two negative laboratory test results. Hospitalization was only indicated for the serious cases requiring a respiratory assistance or special care. A questionnaire was administered by a team of trained physicians, who were responsible to ensure the follow-up of all confirmed cases by phone calls. This questionnaire included questions about sociodemographic characteristics, isolation setting, symptoms and treatment. The recovery statement was based on laboratory data. For asymptomatic patients, the first recovery testing was scheduled for the last day of the isolation period. For symptomatic patients, the first recovery testing was done 3 days after the symptoms disappearance and at least 7 days after onset of symptoms. In both situation, the second recovery testing was done 24 to 48 h after the first negative sample. If the first recovery test was still positive, then the second recovery test was done 7 days later.

The formal criteria for recovery was defined by two successive negative RT-PCR control specimen and recovery time was defined as the period from the date of symptoms onset to the date of a second negative test. (when patients got positive RT-PCR result following false negative result, it was considered as non-effective negative result in our study). After recovery statement, home auto-isolation was advised.

### Laboratory confirmation of SARS-CoV-2 infection

The laboratory testing for SARS by RT-PCR was the laboratory method of reference to confirm cases. Charles Nicolle hospital laboratory which is the national influenzae World Health Organisation (WHO) collaborating laboratory was the reference laboratory at national level. It received all the specimens during the first period of outbreak investigation. Later on, other laboratories performing SARS specific PCR tests were invloved.

Detection of SARS-CoV-2 in nasopharyngeal (NP) specimens was performed at the National Reference Laboratory. RNA was extracted from NP swabs using the GXT NA extraction kit (HainlifescienceGmbh, Nehren, Germany) or the QIAamp Viral RNA Mini kit (Qiagen®, Courtaboeuf, France) following the manufacturers’ instructions. For SARS-CoV-2 RNA detection, the in-house RT-PCR was used according to the Hong Kong protocol (based on two monoplex assays: the N gene RT-PCR as a screening assay and the Orf1b assay as a confirmatory one). With this assay, a positive COVID-19 result is determined when both targets (N and Orf1ab) reach a defined cycle threshold (Ct) of less than 40 [13].

### Statistical analysis

Statistical analysis was performed using SPSS version 17.0 software. Data were expressed as frequencies and percentages for categorical variables and as means and standard deviations (SD) or medians and interquartile ranges (IQR) as appropriate for continuous variables. The median time to recovery was estimated by Kaplan-Meir Methods. Cox proportional hazards regression models were used to estimate factors influencing the recovery time, with the hazards ratio (HR) and its 95% confidence interval (95%CI). Statistical significance was determined at *P* < 0.05.

## Results

### Clinical profile of patients

A total of 1030 patients tested positive for COVID 19 disease were collected between March 2 and May 8, 2020. The mean age was 43.2 years ±18.2, and 526 (51.1%) were female. Ninety seven percent of patients were Tunisians (Table 1) and quarter of infections were imported, mainly from France or Turkey. One hundred and seventy three patients (17.8%) were admitted to the hospital, most of them in a non-intensive care department and only 47 in an intensive care unit (ICU). Sixty six patients (38.2%) received an hydroxy-chloroquine treatment (HCQ). Half of the cases received the HCQ in a hospital setting and the others received it either from a private doctor or as self-medication. Patients who did not require care have been self-isolated at home (55.5%) or in dedicated centers for positive cases (44.5%). One hundred and forty-one patients were healthcare professionals. The source of contamination was the healthcare environment in half of the cases.

**Table 1:** Demographic and clinical characteristics of patients with COVID-19 in Tunisia

Characteristics	N (%)
**Age groups (years)**	
< 15	63 (6.3)
15–34	287 (28.9)
35–54	369 (37.2)
55–74	232 (23.4)
≥ 75	42 (4.2)
**Sex**	
Male	504 (48.9)
Female	526 (51.1)
**Nationality**	
Tunisian	999 (97.0)
Non Tunisian	31 (3.0)
**Medical staff**	
Yes	141 (14.8)
No	809 (85.2)
**Contamination mode**	
Imported contamination	246 (23.9)
Native cases	783 (76.1)
**Hospitalization**	
Yes	173 (17.8)
No	800 (82.2)
**Admission to ICU**	
Yes	47 (4.8)
No	926 (95.2)
**Isolation mode**	
Home isolation	459 (55.5)
Isolation center	368 (44.5)
**Symptoms**	
Yes	602 (74.7)
No	204 (25.3)
**Chloroquine treatment**	
Yes	66 (7.8)
No	779 (92.2)
**TOTAL**	**1030 (100.0)**

*ICU* Intensive care unit

Seventy five percent of patients had symptoms. The most common symptoms were fatigue, cough and fever, which occurred respectively in 51.6, 51.3 and 48.3% of symptomatic patients. Symptoms such as headache, anosmia and digestive signs were also common in those patients (Table 2).

**Table 2:** Distribution of clinical signs among symptomatic patients with COVID-19 in Tunisia

Symptoms	N (%)
Cough	311 (51.6)
Fatigue	309 (51.3)
Fever	291 (48.3)
Headache	210 (34.8)
Anosmia	195 (32.4)
Digestive signs	188 (31.2)
Loss of taste	165 (27.4)
Dyspnea	140 (23.3)
Others signs	129 (21.4)
**TOTAL**	**602 (100.0)**

As of May 8, 2020, a total of 634 (61.6%) patients were recovered, 45 (4.4%) patients were dead and 351 (34.0%) were still being followed up.

### Recovery prognostic factors

**Fig. 1:**
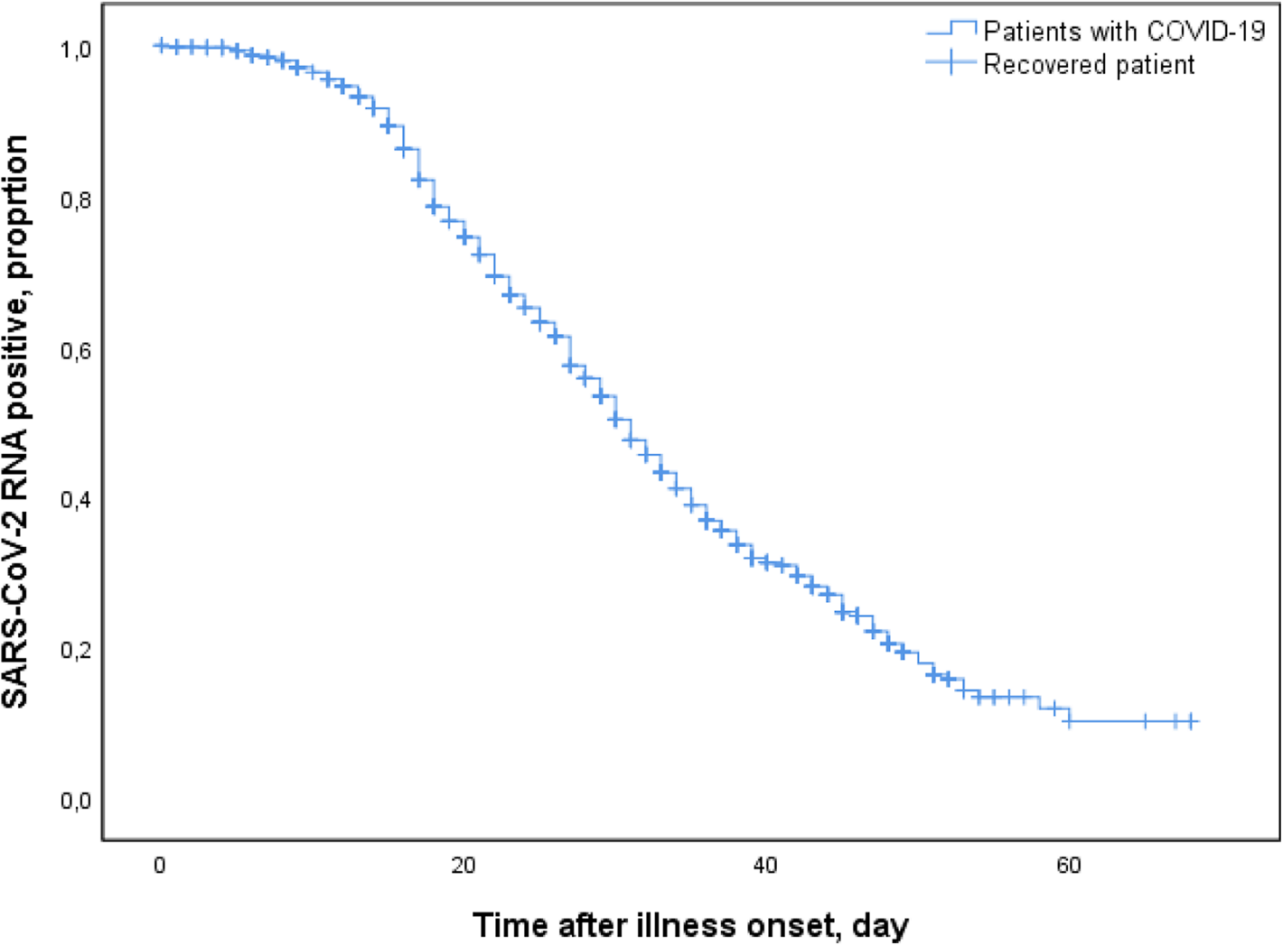
Survival curve showing survival time to recovery from COVID-19 infection

**Fig. 2:**
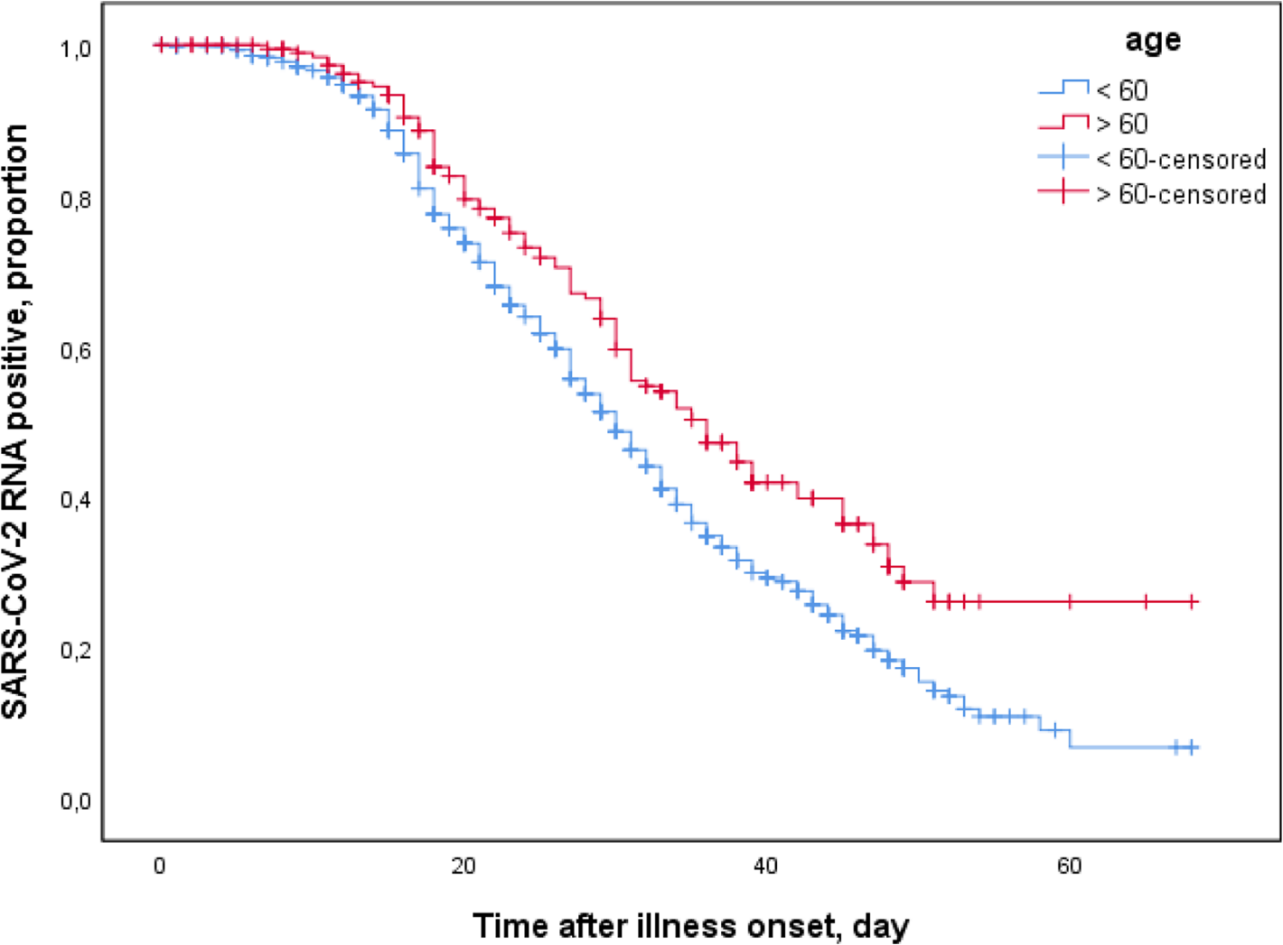
Cumulative proportion of patients with detectable SARS-CoV-2 RNA by day after illness onset between patients aged 60 years and over and patients under 60 years

**Fig. 3:**
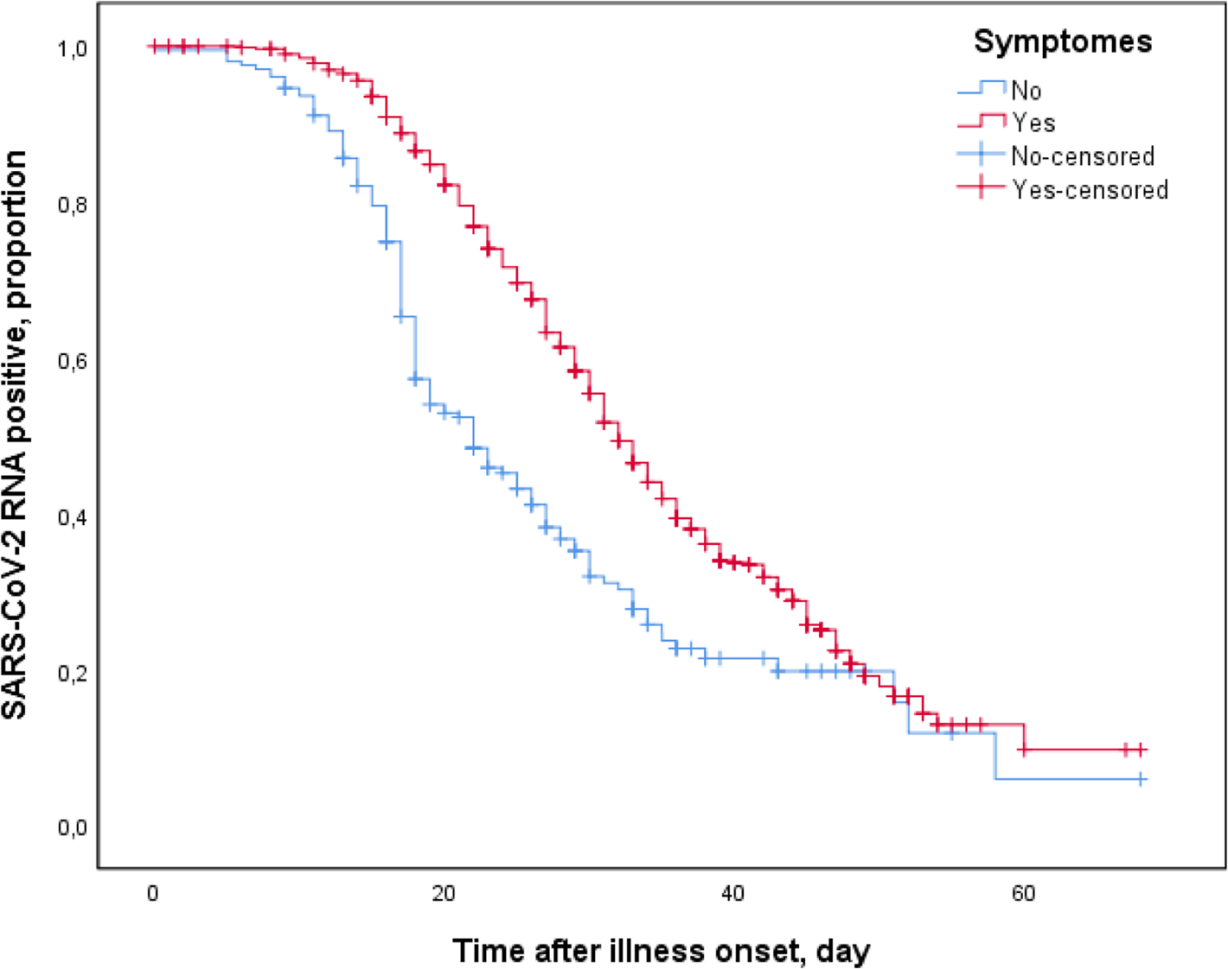
Cumulative proportion of patients with detectable SARS-CoV-2 RNA by day after illness onset between symptomatic and asymptomatic patients

**Fig. 4:**
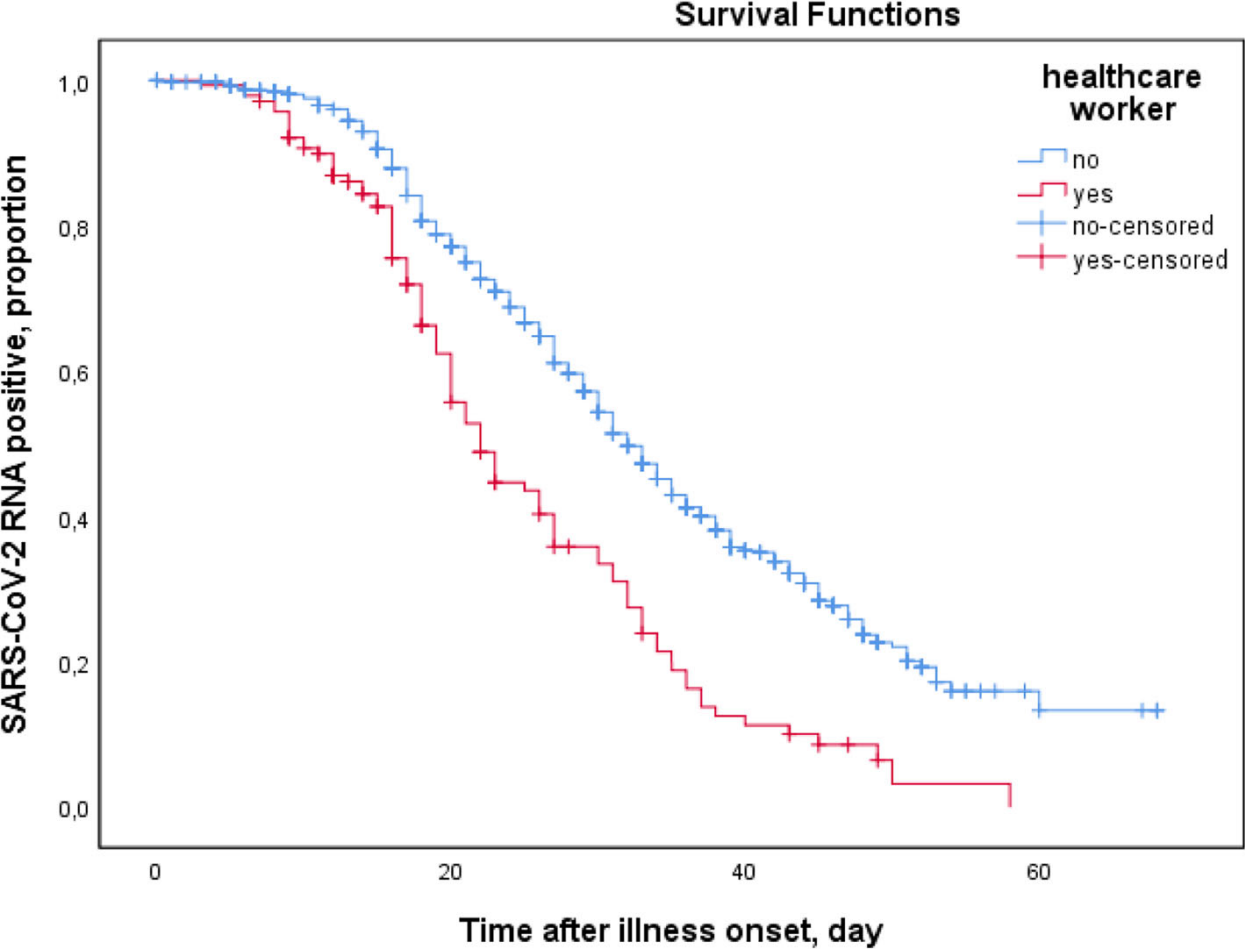
Cumulative proportion of patients with detectable SARS-CoV-2 RNA by day after illness onset between healthcare worker and other patients

**Fig. 5:**
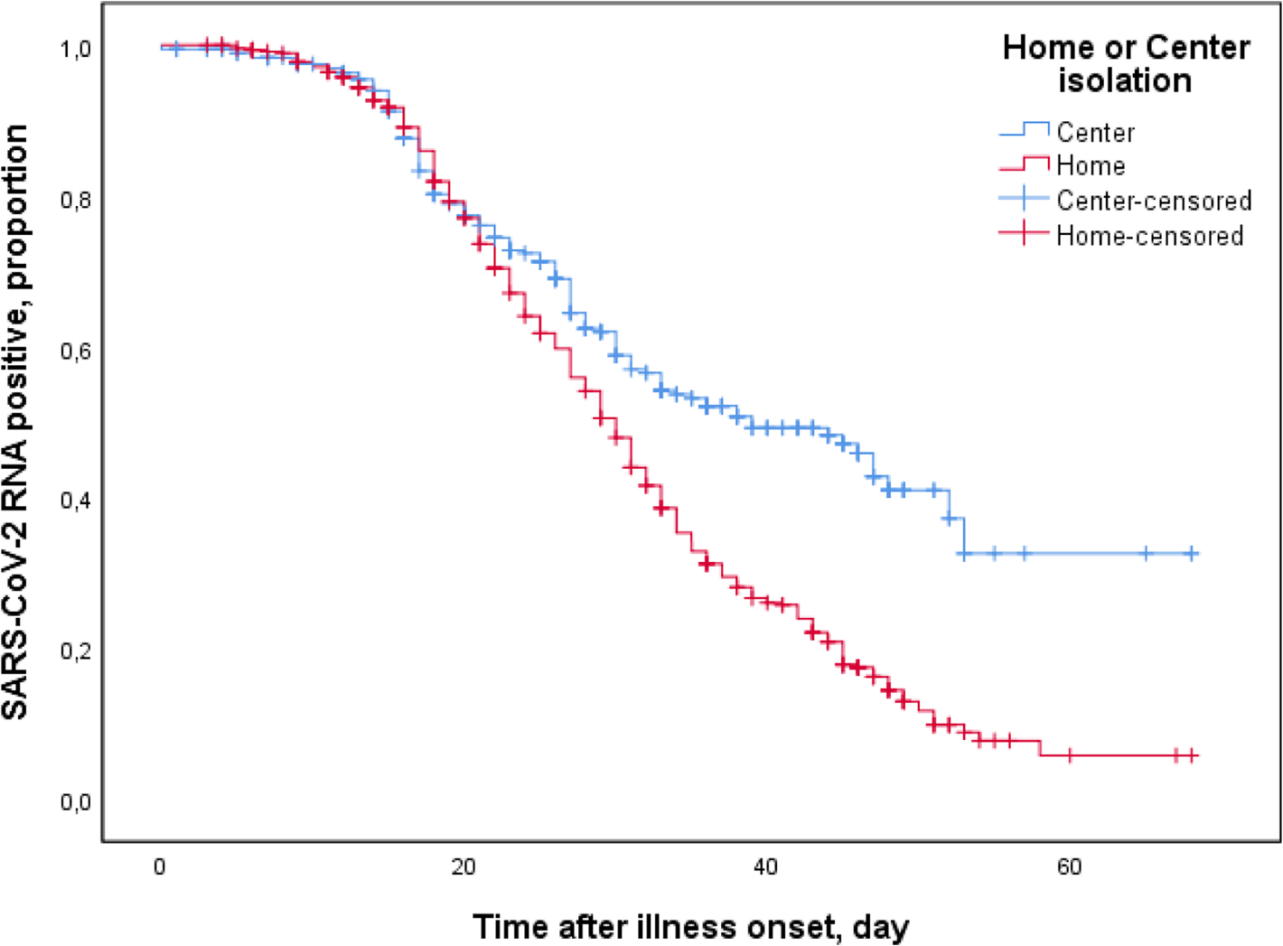
Cumulative proportion of patients with detectable SARS-CoV-2 RNA by day after illness onset between patients in home isolation and patient in dedicated centers for positive cases

Survival analysis was used to analyze the prognostic factors of the disease in patients with COVID-19. It showed that the median duration of illness was estimated to be 31 days (95%Confidence Interval [CI]:[29–32])(Fig. 1). In univariate analysis, older age (Fig. 2), imported cases and being symptomatic (Fig. 3) were associated with a longer duration of illness. Whereas, being a healthcare worker (Fig. 4) and patients in home isolation (Fig. 5) were associated with a shorter duration of illness. Using Cox regression, older age (HR = 0.66, CI:[0.46–0.96], *P* = 0.031) and having symptoms (HR = 0.61, CI:[0.43–0.81], *P* = 0.021) were independently associated with a delay in recovery time. Being a healthcare professional (HR = 1.52, CI: [1.10–2.08], *P* = 0.011) and patients in home isolation compared to isolation centers (HR = 2.99, CI: [1.85–4.83], *P* < 10¯^3^) were independently associated with faster recovery time (Table 3).

**Table 3:** Factors associated with recovery time among patients with COVID-19 in Tunisia

	Univariate Cox regression			Multivariate Cox regression		
	HR	95% CI	***P*** value	HR	95% CI	***P*** value
Male	0.93	0.79–1.09	0.405			
Age > 60 years	0.68	0.55–0.85	0.001*	0.66	0.46–0.96	0.031*
Tunisian Nationality	0.93	0.57–1.50	0.754			
Healthcare worker	1.86	1.52–2.27	< 10¯^3^*	1.52	1.10–2.08	0.011*
Imported infection	0.83	0.69–0.99	0.038			
Home isolation	1.69	1.4–2.06	< 10¯^3^*	2.99	1.85–4.83	< 10¯^3^*
Hospitalization	0.95	0.76–1.18	0.635			
Admission in ICU	0.95	0.71–1.28	0.73			
Symptoms	0.56	0.46–0.68	< 10¯^3^*	0.61	0.43–0.81	0.001*
Chloroquine treatment	1.09	0.82–1.46	0.55			

* Significant *p* values

## Discussion

The main result of our study was the long duration of the disease. It took 31 days from the symptoms onset date, for symptomatic patients, or the laboratory confirmation date, for asymptomatic patients, until the second negative sample.

Our national cohort reached 1030 COVID-19 infected patients. It is a relatively young population with most patients under 55 years of age. In the literature, a mean age between 48 and 55 years was reported [6, 14–17]. For the gender, there was no predominance of men or women. The number of imported cases was higher than the number of native cases until 18th March, 2020. The trend reversal (native cases vs imported cases) was evident from 03/21/2020 which implied an increase in horizontal transmission [5].

Most of cases were mild as evidenced by relatively low ICU admissions and a high number of asymptomatic patients with mild symptoms that do not require treatment in a hospital structure. In fact, the proportion of mild and asymptomatic cases versus severe and fatal cases for COVID-19 infection is currently still unknown. According to a Chinese report publishing all confirmed, suspected, and asymptomatic cases in China, 80% of infections were mild and could recover at home [18]. Concerning clinical presentation of COVID-19 disease, the common symptoms reported by symptomatic patients were cough, fatigue and fever in nearly half of cases and headache in 35% of cases, which was consistent with general symptoms of viral infection. Similar symptoms were described in several case series [6, 15, 19]. One out of three symptomatic patients reported loss of smell and/or taste. This type of disturbance was widely reported in the literature specially among patients with mild and moderate forms of coronavirus disease [20, 21]. A multicenter European study showed that 85.6 and 88.0% of patients reported olfactory and gustatory dysfunctions [22].

The median duration of illness was estimated to be 31 days counting from the day of onset symptoms for symptomatic patients and from confirmation date for asymptomatic patients. This estimation for illness duration was long. In a recent Singapore study, similar results were recorded. It indicated that by day 15 from onset of illness, only 30% of all COVID-19 patients were PCR-negative by nasopharyngeal swab; this rose to 95% by day 33 [23]. According to these results, the duration of viral shedding may extend to a month and sometimes longer for a small group of patients. Current guideline suggested two consecutive negative RT-PCR test results is one of the criteria for hospital discharge or discontinuation of isolation. However, a high false negative rate of viral test was reported and some patients experienced a “turn positive” of nucleic acid detection by RT-PCR test for SARS-CoV-2 after two consecutive negative results, which may be related to the false negative of RT-PCR test and prolonged nucleic acid conversion [24, 25]. The Singapore study indicates also that viable virus was not found after the second week of illness despite the persistence of PCR detection of RNA [23]. It is, then, important to note that traces of virus detected by RT-PCR were not necessarily correlated with the ability of transmission.

In addition, a recent Canadian study demonstrate that infectivity as defined by growth cell culture were most likely between days one and five [26]. These data indicate that even viral RNA detection may persist in some patients, such persistent RNA detection represent non-viable virus and such patients are non-infectious [23]. These findings are consistent with the new WHO recommendations for discontinue transmission-based precautions (including isolation) and release from the COVID-19 care pathway with 10 days after symptom onset, plus at least 3 days without symptoms for symptomatic patients and 10 days after test positive for asymptomatic patients [27]. These new recommendations should be taken in a count to adapt strategy to discontinue isolation of COVID-19 patients in our country.

Cox regression model showed that younger patients recover faster compared to elderly patients. Similar findings were also reported in literature [28, 29]. In fact, many studies found a high proportion of severe cases and fatality rate among elderly patients with COVID-19 [16, 30–33].

Based on the result of this study, being a healthcare worker was showed to be significantly associated to a lower median time to viral clearance after onset of symptoms. This finding could be explained by the fact that the healthcare workers’ monitoring and control was different from that of other COVID-19 patients and that the deadlines for carrying out the control samples were shorter. Indeed, the COVID-19 monitoring and control protocol adopted by the Tunisian health authorities was inspired from the recovery criteria of the European center for disease prevention and control (ECDC) documents [34]. This protocol consists in carrying out a control sample for asymptomatic patients after 14 days from the date of confirmation. For symptomatic ones, if the fever or any other signs persist beyond 14 days, the control sample was postponed for 3 days after the disappearance of the last sign. These deadlines are not respected for healthcare staff involved in the clinical management of COVID-19 patients. Healthcare workers infected with COVID-19 follow a closer control protocol, which may explain the faster time to viral clearance among them.

Results showed that the place of isolation is playing a significant role in survival time to recovery from COVID-19 infection. According to the output of the cox model, COVID-19 patients in self-isolation at home are almost three times more likely to recover faster than those in dedicated COVID-19 centers. In fact, the Tunisian strategy was based in the first phase of the epidemic on the isolation of confirmed COVID-19 cases at home or in dedicated centers and to hospitalize only serious cases requiring special care. Afterwards, given the registration of an increasing number of secondary cases among the close contacts of confirmed patients, the health authorities decided to create specialized centers to isolate COVID-19 patients in order to limit community transmission. The delay in recovery for COVID-19 patients isolated in COVID-19 centers compared to those in self-isolation at home could be explained by the fact that patients do not respect self-isolation inside these centers leading sometimes to mass gatherings. This promiscuity inside the dedicated centers for infected people may thus promote the maintenance of viral load. Symptomatic patients seem also to have slower recovery duration.

This study has some limitations. There was a lack in some clinical information such as comorbidities and vital signs (heart rate, respiration rate, blood pressure etc). In addition, since we used phone interview, information concerning, biological and radiological data were not available for patients who have been hospitalized.

## Conclusions

Isolation of COVID-19 patients in dedicated centers or at home until the negativation of their second control sample was the strategy that Tunisia followed to combat the novel coronavirus. According to the results of our national study, the estimated duration of illness was extremely long, implying the isolation of confirmed COVID-19 cases for long periods. This partly explains the success in controlling the epidemic in Tunisia. However, with current science data, which proves that viral detection by PCR does not equate to infectiousness, we strongly recommend to lighten our isolation strategy in accordance with the new WHO recommendations.

The results we found reinforce guidance that additional control measures must be undertaken to avoid mass gatherings inside isolation centers. Elderly and symptomatic patients need particular attention and closer monitoring.

## Data Availability

The datasets used and/or analysed during the current study are available from the corresponding author on reasonable request.

## Abbreviations

COVID-19: Coronavirus disease
SARS-CoV-2: Severe acute respiratory syndrome coronavirus 2
WHO: World Health Organization
ONMNE: The national observatory of new and emerging diseases
RT-PCR: Real-time reverse transcriptase–polymerase chain reaction
SARS: Severe acute respiratory syndrome
ECDC: The European center for disease prevention and control.
